# TILLING is an effective reverse genetics technique for *Caenorhabditis elegans*

**DOI:** 10.1186/1471-2164-7-262

**Published:** 2006-10-18

**Authors:** Erin J Gilchrist, Nigel J O'Neil, Ann M Rose, Monique C Zetka, George W Haughn

**Affiliations:** 1 Department of Botany, 6270 University Blvd, University of British Columbia, Vancouver, BC, V6T 1Z4, Canada; 2 Department of Medical Genetics, University of British Columbia, Vancouver, BC, V6T 1Z3, Canada; 3 Department of Biology, McGill University, Stewart Building N5/16, 1205 Avenue Docteur Penfield, Montreal, QC, H3A 1B1, Canada

## Abstract

**Background:**

TILLING (Targeting Induced Local Lesions in Genomes) is a reverse genetic technique based on the use of a mismatch-specific enzyme that identifies mutations in a target gene through heteroduplex analysis. We tested this technique in *Caenorhabditis elegans*, a model organism in which genomics tools have been well developed, but limitations in reverse genetics have restricted the number of heritable mutations that have been identified.

**Results:**

To determine whether TILLING represents an effective reverse genetic strategy for *C. elegans* we generated an EMS-mutagenised population of approximately 1500 individuals and screened for mutations in 10 genes. A total of 71 mutations were identified by TILLING, providing multiple mutant alleles for every gene tested. Some of the mutations identified are predicted to be silent, either because they are in non-coding DNA or because they affect the third bp of a codon which does not change the amino acid encoded by that codon. However, 59% of the mutations identified are missense alleles resulting in a change in one of the amino acids in the protein product of the gene, and 3% are putative null alleles which are predicted to eliminate gene function. We compared the types of mutation identified by TILLING with those previously reported from forward EMS screens and found that 96% of TILLING mutations were G/C-to-A/T transitions, a rate significantly higher than that found in forward genetic screens where transversions and deletions were also observed. The mutation rate we achieved was 1/293 kb, which is comparable to the mutation rate observed for TILLING in other organisms.

**Conclusion:**

We conclude that TILLING is an effective and cost-efficient reverse genetics tool in *C. elegans*. It complements other reverse genetic techniques in this organism, can provide an allelic series of mutations for any locus and does not appear to have any bias in terms of gene size or location. For eight of the 10 target genes screened, TILLING has provided the first genetically heritable mutations which can be used to study their functions *in vivo*.

## Background

*Caenorhabditis elegans *is a well-established model system (reviewed by Hodgkin [1]) that is increasingly being used for genetic and molecular investigations into conserved biological processes, including those involved in human disease [2-5]. Although simple in structure, *C. elegans *is comparable to higher animals in development and forms most of the major tissue types that are important to vertebrate physiology. Indeed, in a comparison of 18,452 *C. elegans *protein sequences against human EST databases, 83% (15,344 sequences) of the *C. elegans *sequences were found to have human homologues [6].

Because the sequence of the complete *C. elegans *genome has been available since 1998, bioinformaticians have been presented with ample opportunity to mine the data, and a plethora of genomic and proteomic information is accessible to researchers wishing to build upon this information [7]. Powerful *in silico *techniques have also been developed for the analysis of genome sequence information and are used in the prediction of gene function, expression and interaction [5,8,9]. Despite the exciting possibilities flowing from these studies, the testing of predictions made *in silico *relies largely on the existence of efficient reverse genetic approaches that target specific genes or classes of genes *in vivo. In vitro *techniques such as yeast two-hybrid analysis [10] and microarray analysis [11] have also been used to generate an abundance of valuable data about gene expression and protein interactions but, like the data generated *in silico*, these data need to be verified *in vivo*.

*C. elegans *has approximately 19,800 protein-coding genes and 12,000 of these have been conserved over the 100 million years since this species has diverged from the related nematode *Caenorhabditis briggsae*, indicating that they are likely important functional genes [12]. In spite of this fact, however, only about 3,400 genes in *C. elegans *have mutant alleles available for genetic and biochemical analysis to ascertain their function and importance [13]. High-throughput reverse genetics is an ideal way of generating mutations in the remaining 16,400 genes and several such approaches have been developed for the nematode each of which has advantages and drawbacks that affect the applicability or efficiency of the technique as a tool for probing gene function on a genomic scale.

Currently, the most efficient and popular method to disrupt the activity of a gene in *C. elegans *is the technique of RNA interference (RNAi) [14]. Large-scale RNAi screens have demonstrated that the function of a diverse population of genes with roles in many biological processes can be disrupted by the injection of double-stranded RNA (dsRNA) directly into the gonad [15], by soaking the nematodes in a dsRNA solution [16], or by feeding the nematodes bacteria expressing dsRNA [17,18]. These same studies, however, have also documented that the phenotypes resulting from the RNAi treatment often depend on the method of delivery. In addition, the RNAi technique cannot replace classical genetic analysis because the phenotypic effects are transient and not heritable, making classical genetic interaction studies impossible.

Another effective reverse-genetic technique that is being used successfully in *C. elegans *is mutagenesis with trimethylpsoralen and ultraviolet radiation (TMP/UV) followed by detection of gene knockouts by PCR. This is currently the method of choice for obtaining heritable loss-of-function mutations in *C. elegans *but there are also drawbacks to this approach. First, the limitations of the detection method necessitate using a high dosage of mutagen which requires multiple rounds of outcrossing to remove accompanying background mutations. In addition, missense alleles cannot be isolated and large deletion events may result in the loss of function from more than one locus simultaneously. Finally, although the reason for this is unclear, mutations in certain genes have been more difficult to obtain than in others.

Transposon-insertion mutagenesis is another tool that is available to the *C. elegans *community [19,20] but it shares many of the limitations discussed for the previous techniques in addition to some that are specific to this approach such as the fact that small genes are less likely to be targets of transposon insertion and certain regions of the genome may vary in the frequency at which transposons insert. The mutagenic effect of Tc1 insertions can also sometimes be circumvented by innate compensation mechanisms that allow spicing around the transposon.

A recently reported study of biolistic transformation in *C. elegans *indicates that homologous recombination of introduced DNA is also possible in this species [21] but, in spite of the potential of this technique to provide the long-sought ability to perform site-directed mutagenesis in *C. elegans*, the low success rate and the fact that an elaborate microparticle bombardment set-up is required, make it unlikely that this procedure will soon become efficient enough for high-throughput reverse genetics.

**Figure 1:**
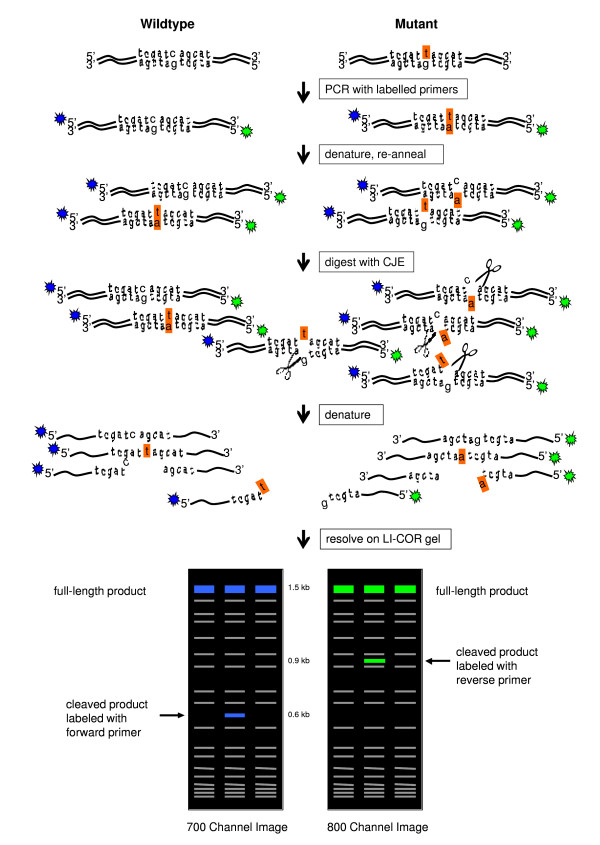
Overview of the TILLING procedure. Pooled DNA is amplified using fluorescently tagged, gene-specific primers. The forward and reverse primers are labelled with different fluorophors that label both ends of the fragment. The amplified products are denatured by heating and then allowed to cool slowly so that they randomly re-anneal. Heteroduplex molecules form when mutant and wild-type PCR products anneal together, and these then become targets for a single-strand-specific nuclease found in Celery Juice Extract (CJE). The nuclease cleaves these heteroduplex fragments at one of the two strands, 3' to the site of the mismatch in the DNA. The PCR products that retain one of the labelled primers can then be detected on polyacrylamide denaturing LI-COR gels. Individuals with a mutation in the gene of interest are identified by the smaller cleavage fragment seen on the gel as well as the wild-type product. Because the nuclease cleaves either of the two strands randomly, cleavage products can be detected in both the IRD700 and IRD800 channels of the gel image. The position of the mutation within the PCR amplicon can be calculated from the size of the two fragments carrying the forward, IRD700-labeled primer, and the reverse, IRD800-labeled primer. Grey bands on the gel are thought to result from partial PCR products and aid in sizing of mutant bands.

As a result of drawbacks in currently used reverse genetic techniques, the pace of research into biological processes in *C. elegans *is still largely dictated by the probability of obtaining a mutant of any given gene and, thus, new techniques are needed to complement those previously described. TILLING (Targeting Induced Local Lesions in Genomes) is a relatively novel reverse genetics technique based on the use of a mismatch-specific enzyme that will identify mutations in any target gene through heteroduplex analysis [22]. The technique involves PCR amplification of a target gene or region of DNA using fluorescently labelled primers, followed by digestion with an enzyme that specifically cleaves at the site of a mismatch such as that induced by ethylmethanesulfonate (EMS) mutagenesis (see Figure 1). The sizes of the cleavage fragments resolved on polyacrylamide gels reveal the approximate position of the mutation within the amplicon. We report here on a pilot project to test the use of this technology in *C. elegans*: we have constructed and arrayed a mutagenised population and used it to isolate mutations in 10 different genes. On the basis of these data we conclude that TILLING is as effective and cost-efficient in *C. elegans *as it has been shown to be in other species in which it has been tested [23-29].

## Results and Discussion

### Efficiency of TILLING in C. elegans

To determine whether TILLING represents an effective reverse genetic strategy for *C. elegans *we generated and arrayed an EMS-mutagenised population of approximately 1500 individual animals (see below) and screened for mutations in 10 genes varying in size from 788 base pairs (bp) to 9112 bp. A region of approximately 1500 bp from each gene was examined and a total of 71 novel mutations were identified by TILLING, thus providing multiple mutant alleles for each gene (Table 1). Some of the mutations we identified are predicted to be silent, either because they are in non-coding DNA or because they affect the third bp of a codon which does not change the amino acid encoded by that codon. However, 59% of the mutations we identified are missense alleles resulting in a change in one of the amino acids in the protein product of the gene, and 3% are nonsense alleles resulting from the insertion of a premature stop codon into the coding region of the gene, or in the elimination of a conserved splice junction site. These data demonstrate the efficacy of TILLING in *C. elegans*.

### Comparison of forward and reverse genetics with EMS mutagenesis

**Figure 2:**
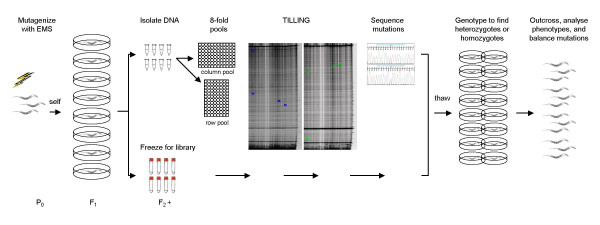
Outline of *C. elegans *TILLING procedure. Animals are mutagenised with EMS, picked individually to plates, and allowed to self. One third of the worms are used for DNA and the remaining two thirds are frozen for future analysis. DNA is pooled 8-fold to reduce time and expense. TILLING is performed in order to determine which individuals carry mutations in the gene of interest. Mutations are sequenced and individuals from lines carrying mutations that have an effect on the gene product are thawed and genotyped to isolate heterozygous or homozygous mutants.

A *C. elegans *population was constructed for this TILLING project using the mutagen EMS (Figure 2). EMS was chosen because it has been shown to be an effective mutagen in this species and because it is known to generate primarily single bp mutations which can be identified using TILLING [23,30-34].

A survey of Wormbase [7] was performed in order to examine the type of molecular lesion induced by EMS in *C. elegans *to ensure that the majority of these mutations are, indeed, small intergenic lesions of the type that can be identified by TILLING (see Additional File 1 for a list of alleles). Two hundred and thirteen alleles from 51 different genes whose molecular sequence was known were selected randomly from the database. All of the mutations were reported to be identified in screens of EMS-treated animals. Ninety three percent of the 213 alleles examined from Wormbase were found to be single bp mutations. Eighty seven percent were G/C-to-A/T transitions, six percent were other single bp mutations, and seven percent of the mutations reported were deletions that ranged in size from 88 bp to 2.3 kb.

In our TILLING experiment, 68 of the 71 independent mutations identified (96%) were G/C-to-A/T transitions and the remaining three mutations were A/T-to-T/A or G/C-to-T/A transversions (Table 2). This percentage of G/C-to-A/T transitions is significantly higher than that found in forward genetic screens and probably reflects the fact that many EMS-induced lesions are not identified using classical genetic screens because they do not have an effect on phenotype. The frequency of transversions seen with TILLING in our screen is similar to the frequency found in forward genetic screens using EMS in *C. elegans *(6%), but significantly lower than the number of transversions mutations reported in a small *Drosophila *TILLING project (16%) [29], and significantly higher than the number found in *Arabidopsis *where greater than 99% of mutations sequenced were G/C-to-A/T transitions [23].

Three of the mutant strains we generated in this study, CN579, CN1162 and CN1574 carried two point mutations within the target gene. In each case, one of the second-site mutations was in non-coding DNA and so presumably would not affect the phenotype of the animals carrying the linked mutation. Second-site mutations have been reported in forward genetic screens of EMS-treated worms as well. A case in point is the molecular analysis of *unc-52 *mutations and intragenic suppressor alleles [35]. Two of the 19 mutations sequenced in the *unc-52 *study carried a second site mutation less than 400 bp upstream of the primary mutation. One of these second-site mutations was a single bp transition, and the other was a 311 bp deletion. In both cases, the second-site mutations were not found to affect the phenotype of the animals carrying them. Second-site mutations have also been found in EMS screens of yeast [33] and *Drosophila *[34], and so presumably reflect some common DNA repair mechanism that is induced upon EMS damage.

Deletions are another type of mutation that has been reported in forward genetic screens using EMS in several different species [30,32,34]. In *C. elegans *approximately 7% of all mutations from forward genetic screens of EMS-treated animals are deletions (Additional File 1). We did not identify any deletions in this TILLING project but we are confident (based on previous studies [23,36]) that if EMS does generate this type of mutation in *C. elegans*, TILLING will be able to detect these events as well as the single base pair changes that are more common. The reason that deletions have been identified more frequently in forward genetic screens than in our reverse genetic screen is probably because these mutations are much more likely to produce a phenotype than the single bp mutations more commonly produced by EMS.

### Mutagen dose and mutation rate

The dose of EMS used for our TILLING project (0.025M) was lower than that used in many forward genetic screens because studies have shown that this lower dose simplifies the identification of mutant phenotypes caused by the gene of interest while limiting confounding background phenotypes or lethality [37]. In two strains, however (CN843 and CN1643), we identified mutations in two different genes in the same strain. While this might seem to indicate a very high overall mutation frequency, we do not believe that this is the case since the mutagenised animals seem healthy and fertile, and since the overall mutation rate was calculated to be one mutation every 293 kb (71 mutations in 14225 bp of DNA from 1464 animals). This is not significantly higher than the rate of one mutation in 300 kb seen for TILLING in *Arabidopsis *[23], and is lower than the rate of one mutation in 156 kb reported for *Drosophila *[29]. Hence, the 0.025M dose of EMS appears to be an adequate dose for TILLING based on comparison with these other systems in which this technique is currently being used.

### Effects of mutations identified through TILLING

The spectrum of mutations identified through forward and reverse screens using EMS, although similar at the level of DNA sequence is much different when the effects of the mutations are compared. Of the 194 single bp mutations we analysed from Wormbase (Additional File 1) 50% resulted in missense alleles and the remaining 50% in nonsense or splice junction mutations. In our TILLING screen, because the selection of mutants was not based on phenotype, 38% of the mutations we identified are predicted to be silent. These include mutations in introns and intergenic regions and mutations that alter the third bp of a codon such that it still encodes the wildtype amino acid. The majority of the mutations we identified (59%) were missense alleles that alter the amino acid sequence of the protein encoded by the target gene (Table 2). Of the 42 missense mutations identified in our screen, 17 of these may not have a significant effect on phenotype since the amino acid mutated was replaced by an amino acid of similar charge and polarity, but the 25 remaining missense mutations are predicted to significantly affect the structure of the protein product of the target gene by changing the charge or hydrophobicity of this region of the protein.

**Figure 3:**
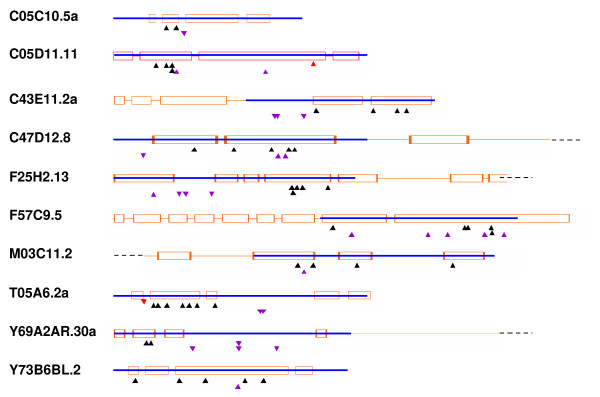
Gene models depicting the distribution of different types of mutations within the genes. The figure was designed from PARSESNP [42] output files. Blue lines indicate the extent of the amplified region that was used for TILLING. Orange open boxes denote exons. Purple up arrows indicate a change in the DNA sequence that does not affect the amino acid product. Purple down arrows indicate a change in non-coding DNA. Black up arrows indicate a change that induces a missense mutation in the predicted protein product. Red up arrows indicate a premature stop codon or splice junction error.

Two of the mutations identified in our TILLING screen, *mel-32 *C05D11.11*(vc68) *and *cki-2 *T05A6.2*(vc39)*, are predicted to result in a complete loss-of-function, or null, phenotype because they truncate the protein product of the gene. One of these introduces a premature stop codon into the third exon of gene *mel-32 *C05D11.11, and the other is a splice junction mutation that eliminates the splice donor site of first intron of the gene *cki-2 *T05A6.2 (Figure 3). The proportion of putative null mutations identified in our screen was 3% which is not significantly different than the frequency of 2% seen in the *Drosophila *TILLING study published [29] or than the 5% reported from the much larger *Arabidopsis *TILLNG project [23]. This frequency is higher than would be expected using other chemical mutagens in *C. elegans *such as ENU [38] which cause a different spectrum of mutagenic events that are more likely to result in missense than nonsense mutations.

### Pooling and library construction

A frozen library of approximately 1500 individual EMS-treated lines of *C. elegans *was constructed for this study (Figure 2), and DNA was isolated, purified, and arrayed in pools of eight as has been shown to work for TILLING in other diploid species such as *Arabidopsis thaliana *[22], *Lotus japonicas *[24], *Zea maize *[27] and *Brassica oleracea *[Gilchrist and Haughn, unpublished]. Purification of the DNA was found to be necessary both because the TILLING reactions did not work on unpurified DNA samples and because accurate quantitation of the samples is essential before pooling so that DNA from mutant animals is sufficiently represented in the 8-fold pools [27]. Both 4-fold and 12-fold pools were tested in *C. elegans *in order to confirm that 8-fold pooling would be efficient (see Materials and Methods for details). With 4-fold pooling all of the mutant bands were detected, as they were with the 8-fold pools, whereas with 12-fold pooling only a subset of the mutant bands were seen on the TILLING gel. Although 10-fold pools might be possible in *C. elegans *given that the genome size of this organism is slightly smaller than *Arabidopsis*, the construction of libraries for screening in 96 or 384-well plates dictates that pooling is only efficient in multiples of eight or 12, and since mutations are missed with 12-fold pooling in *C. elegans*, libraries constructed for this TILLING study were pooled in 8-fold aliquots.

Our first library consisted of 696 rather than the planned 768 (8 × 96) mutagenised worms because the DNA quality in 72 of the 768 lines established was too poor to be used for TILLING. Only 8-fold column pools were constructed for this library and when a mutation was detected in a column pool well, each of the eight individuals that made up that pool was examined by TILLING in order to determine which strain carried the mutation. For the second library of 768 animals, however, both 8-fold row pools and 8-fold column pools were constructed and screened. The DNA from individual worms was arrayed in 12, eight-by-eight grids so as to simplify pooling in either direction. Then the DNA was pooled by combining samples from one row or one column into a single pool, resulting in a total of 96 pools. The identity of the strain carrying a mutation detected on the TILLING gels was computed automatically by cross-referencing the data from the row and column pools.

In theory, the method used for screening library #1 only required an average of 120 reactions: one 8-fold column pool, plus eight individuals for each of three mutations detected (24 additional reactions). However, the 24 reactions done to determine which individual from a pool carried the mutation had to be set up manually and often needed to be repeated in order to ensure that all of the reactions worked. Thus, this pooling strategy was more time-consuming and often required almost as many TILLING reactions as the screening of both row and column pools simultaneously. In addition, the row and column pool strategy allowed false positive bands to be excluded from our study since a mutation was never followed unless it appeared in at least one channel in both row and column pool gels.

### Selection of targeted regions

Mutations identified through TILLING are randomly distributed in the genome, thus making it possible to target genes of any size and at any location [23]. The average size of the region that is tested in one TILLING screen is usually about 1500 bp and this is the size that was used for most genes in this study (Table 1). For the gene C05C10.5 whose genomic sequence is only 788 bp we designed primers that amplified a region of approximately 1200 bp to avoid screening excess intergenic DNA upstream or downstream of the locus where mutations would have a higher probability of being silent. In addition, for gene *mus-81 *C43E11.2, the primer sets that we designed to amplify a product of 1500 bp gave multiple amplification bands when used with our standard PCR conditions. Thus we were forced to use a primer set that amplified a smaller region in order to obtain a single, clean PCR product from this locus.

For genes that are much larger than 1500 bp, two approaches have been used for TILLING in other systems. The web-based programme, CODDLE [39] was originally designed for use in the *Arabidopsis thaliana *TILLING project and assists researchers in designing primers to select regions of a gene of interest that are most likely to provide loss-of-function or deleterious alleles. Researchers also have the option of requesting that CODDLE design primers that will amplify a fragment within a specific region of the gene in which they are most interested, for example, a specific domain that is known to interact with another gene or protein. A different approach was used for a recent *Drosophila melanogaster *TILLING project [29]. In this study, multiple primers were designed to amplify overlapping fragments of a gene so that the entire gene could be screened by TILLING. For our TILLING project in *C. elegans*, as mentioned previously, the average amplicon size was 1500 bp and primers were designed to amplify the region of the gene predicted by CODDLE to be most susceptible to EMS-induced mutations. Primer sequences used in this study are listed in Table 3. For *C. elegans*, where the average gene size is 3000 bp and introns are generally small [40], TILLING should prove to be even more efficient than in other species where larger genes and intervening sequences are the norm. Indeed, 62% of mutations recovered in this *C. elegans *screen are predicted to have an effect on the protein product (Table 2), although long-term studies are required to determine exactly how many of these mutations will result in a mutant phenotype.

### Identification of individual mutant animals

Each F_1 _mutagenised line was frozen individually in order to ensure that identified mutations could be recovered even if a sample was frozen and thawed multiple times (see Figure 2). Except in the case of deleterious mutations that affect viability or reproduction, each mutant allele that is heterozygous in the F_1 _parent is expected to be present in 3/4 of the progeny of this individual. If the mutant allele is recessive lethal, then the frequency of progeny carrying this allele should be 2/3. In this study we were able to recover individual descendants of F_2 _worms carrying the mutant alleles in 19 of the 20 strains thawed for testing. The remaining strain proved to be a false positive isolated from our screening of our first library. The probability of this type of error occurring with our current methodology of screening both row and column pools is very low.

**Figure 4:**
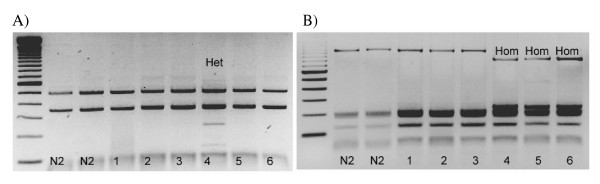
Restriction enzyme digests of DNA from heterozygous and homozygous mutants. A) CAPS analysis of sibling lines for CN646 *htp-3(vc1) *using the restriction enzyme Taq1. The lanes labelled N2 are wildtype controls. Lane marked 4 exhibits additional bands when digested with this enzyme indicating this line is heterozygous for the *vc1 *mutation. B) CAPS analysis of sibling lines for CN711 *mdf-2(vc15)*, using the restriction enzyme Hinf1. The lanes labelled N2 are wildtype controls. Lanes marked 4, 5 and 6 show additional cleavage bands and are missing the wildtype band indicating that they are homozygous for the *vc15 *mutation.

There are several methods that can be used to follow the segregation of point mutations in F_2 _and subsequent populations. We used three different strategies for comparison in this study: TILLING, cleaved amplified polymorphic sequences (CAPS) [41], and direct sequencing. TILLING was the most expensive and labour-intensive method because of the requirement to purify the extracted DNA for PCR and because detection of homozygous individuals necessitated duplexing the DNA from the putative mutant with wildtype DNA (since fragments are only cleaved with CJE if there is a mismatch in the DNA). In addition, multiple rounds of TILLING were sometimes necessary if one of the two reactions (duplexed and non-duplexed DNA) failed, because in such a case the zygosity of the animal in question could not be determined. CAPS was tried if the PARSESNP programme ([42] indicated one or more restriction enzyme polymorphisms between the mutant and wildtype sequences (Figure 4). Sixty of the 71 mutations we identified (85%) in this screen are of this type. For the samples where there were no restriction enzyme polymorphisms either TILLING or direct sequencing was used to detect mutant individuals because of time constraints, although studies have shown that the derived cleaved amplified polymorphic sequences (dCAPS) technique works well and would be a less expensive method for following mutations in the long-term [43].

Results from direct sequencing of DNA from F_2 _descendants of mutant animals showed that the mutation of interest was present in an average of two out of three of the thawed progeny (43 out of 64 samples), although this varied from a low of one out of eight with F25H2.13*(vc9)*, and a high of 13 out of 16 with *htp-3 *F57C9.5*(vc2)*. Both of these alleles with non-typical segregation patterns are missense mutations and, although the *vc2 *allele does not have an obvious phenotype in homozygous mutants, we speculate that it may be incompatible with the wildtype allele of this locus since heterozygous individuals carrying both mutant and wildtype alleles together were never recovered. The high frequency of homozygous *vc2 *mutants compared to wildtype individuals may just be a statistical anomaly or may indicate that the mutant protein confers some type of fitness advantage upon animals carrying it.

Two other mutations, F25H2.13*(vc10) *and *mel-32 *C05D11.11*(vc11)*, were apparently homozygous in the parent F_1 _strains in which these alleles were detected because all of the thawed progeny from the F_2 _plates were found to be homozygous for these mutations. All other mutations were heterozygous in the F_1 _generation, as expected, because EMS-induced damage in *C. elegans *usually occurs in either the P_0 _egg or the P_0 _sperm before fertilization. Neither of the homozygous mutations was present as a background mutation in the wildtype strain used for mutagenesis, or in any of the other mutant strains whose DNA was sequenced. The F_1 _homozygosity of *vc10 *and *vc11 *may be the result of an early mitotic crossover or other heritable event that occurred in the developing zygote rather than in the maternal germ cells, during or after EMS mutagenesis. The fact that these mutations were detectable at all using TILLING is curious since a DNA mismatch is needed for cleavage with CJE. In the pooled samples, adequate wildtype DNA from other samples would have been amplified and paired with the mutant DNA for cleavage to occur, but when testing the DNA from the individual F_1 _animals no wildtype DNA should have been present since we did not detect any wildtype progeny from these animals. It is possible that only the germ cells of the F_1 _animal carried the mutations identified in our screen and that wildtype DNA from the somatic cells of this animal was sufficient to allow for cleavage with CJE when amplified using PCR. It is also possible that some wildtype DNA contamination was present in these reactions and was amplified along with the homozygous DNA from the mutant. The fact that the cleavage bands were very faint on the TILLING gels is consistent with either of these ideas.

An additional polymorphism in gene F25H2.13 was identified when sequencing other alleles of this gene and found to be homozygous in all TILLING strains and in the N2 P_0 _strain utilised for mutagenesis in this study, making it clear that this strain is different from the N2 strain used in the *C. elegans *genome sequencing project. Although this polymorphism induces an amino acid change in the protein sequence, it has no obvious effect on gene function since animals carrying the polymorphism are seemingly wildtype.

### Analysis of mutants identified through TILLING

Some of the loci chosen as candidates for this pilot project were genes that are thought to play roles in chromosome segregation, recombination or genome maintenance. In some cases, RNAi constructs of these genes had been shown to induce varying phenotypes, and we reasoned that null and missense alleles of these targets would allow us to better identify the true function of these genes. In other cases, the genes we targeted had no known RNAi phenotype, but had been implicated in meiotic functions through two-hybrid or bioinformatics studies.

Examination of the sequence of the 71 alterations shown in Table 2 revealed that 27 of the changes resulted in no amino acid change (silent mutations) and these strains are unlikely to have visible phenotypes. Of the remaining 44 mutations resulting in amino acid changes, 24 affected charged or conserved residues. These are the alleles that are most likely to affect the functioning of the gene product and thus most likely to have phenotypic changes. We have done preliminary phenotypic analysis on 25 of the TILLING alleles we identified and observed phenotypes for 16 of these alleles. Although further studies are needed to confirm these data, the TILLING alleles reported here clearly allow characterisation of these genes in a manner that was not previously possible.

#### *mel-32 *C05D11.11

The gene, *mel-32 *C05D11.11 was selected simply as a control because many mutations at this locus have been identified through forward genetic screens and sequencing of these indicates that most of the amino acids in the encoded protein are essential for normal gene function [46]. Mutations in this gene result in a maternal-effect lethal phenotype (Mel). The homozygotes are viable and fertile, but produce eggs that fail to hatch. The putative null allele isolated by TILLING (*vc68*) does indeed have a Mel phenotype. The three remaining missense alleles are currently being examined, although preliminary evidence indicates that one of them (vc11) is a conservative change that does not appear to confer any mutant phenotype.

#### C05C10.5

A gene name has not yet been assigned for this locus (indicated by * in Table 1). Little is known about this gene or the function of the gene product. RNAi treatment produces variable results ranging from embryonic lethality to high incidence of males (Him). We have two TILLed alleles (*vc21 *and *vc40) *that were isolated as heterozygotes and are being maintained as such. As a consequence we have not yet determined whether or not these alleles will cause a mutant phenotype.

#### *mus-81 *C43E11.2

We have isolated the first genetic mutations in this gene through TILLING. Three of the missense mutations (*vc42, vc46 *and *vc47*) have a radiation-sensitive (Rad) phenotype. The mutant strains, which can be maintained as homozygotes, vary in the severity of their response to radiation, and thus will provide valuable resources for dissecting the molecular characteristics of this gene. Homozygous *mus-81(vc46) *animals are severely radiation sensitive, while animals carrying either *mus-81(vc42) *or *mus-81(vc47) *exhibit less severe phenotypes. These phenotypes are consistent and continue to segregate with the molecular mutations even after multiple outcrossings.

#### *xpf-1 *C47D12.8

This gene is the *C. elegans *orthologue of the essential nucleotide excision repair gene *XPF/ERCC4 *[48]. We have identified three mutations in this gene (*vc18, vc19 *and *vc67*). All of these are missense alleles, and we have determined that two of them (*vc19 *and *vc67*), have a Rad phenotype as would be predicted. None of the alleles is lethal since they can be maintained as homozygotes.

#### F25H2.13

A gene name has not yet been assigned for this locus (indicated by * in Table 1), but it is predicted to encode a DEAD helicase closely related by sequence to DOG-1. RNAi treatment reveals no detectable phenotype and the deletion allele (*tm1866*) is listed in Wormbase [7] as homozygous viable. We have identified five new missense alleles through TILLING, and strains carrying these alleles are also viable although they appear to have reduced brood sizes.

#### *htp-3 *F57C9.5

Excellent antibodies are available for studying the protein product of this HIM-3 paralogue *in vivo*, but the deletion allele *htp-3*(*gk26*) is associated with a complex rearrangement that includes a wild-type copy of the gene, making phenotypic analysis impossible. RNAi experiments revealed that the animals exhibit no phenotype when the worms are fed a dsRNA construct for *htp-3 *F57C9.5 [44], but injection of the dsRNA results in severe embryonic lethality as a consequence of chromosome nondisjunction [45]. We have isolated five new missense alleles of this gene by TILLING, and observed varying levels of embryonic lethality that segregate with the mutant allele for three of these mutations (*vc1, vc75 *and *vc77*).

#### M03C11.2

A gene name has not yet been assigned for this locus (indicated by * in Table 1), but the gene is a member of the DEAD helicase family related to DOG-1. RNAi treatment produces arrested embryos, and a deletion allele (*tm2188*) has been shown to be sterile, but we have not yet determined whether any of the missense alleles we have identified by TILLING have phenotypes.

#### *cki-2 *T05A6.2

The knockout allele of this gene *cki-2*(*ok741*) causes sterility in homozygous animals as does the TILLING mutation *cki-2*(*vc39*) which affects a conserved splice junction site. The strain is easily maintained as a heterozygote, however, and can used for genetic analysis in this way.

#### *mdf-2 *Y69A2AR.30

This gene was studied previously using RNAi and shown to have reduced brood size and increased incidence of males [47]. Using TILLING, we have successfully identified the first genetic mutation in this gene. The *mdf-2(vc15) *mutation can be maintained homozygously despite exhibiting reduced brood size and high incidence of males, and thus provides a valuable tool for the study of metaphase to anaphase checkpoint signalling.

#### *htp-2 *Y73B6BL.2

This gene encodes a paralogue of HIM-3 and has been shown to play a role in meiotic function. RNAi treatment produces a Him phenotype. We have five TILLed alleles of this locus that are viable as homozygotes and for which we have observed no obvious phenotype.

One of the major advantages of TILLING is that it can not only identify null mutations which eliminate the function of the gene product entirely, but also missense alleles that result in a partial loss or change of gene function and which can allow disruption of specific domains within a gene and are especially useful for suppressor screens which can be used to identify interacting genes. The reverse genetic techniques that are presently being used in *C. elegans *are all more likely to result in complete-loss-of-function alleles which, if the effect is lethal or detrimental, may limit the analysis that can be done. With TILLING, however, it is possible to use missense mutations in different regions of the gene for the dissection of multiple functions and interactions of a given gene product. While the point mutations that TILLING identifies can result in complete loss-of-function alleles that are as effective as deletions in knocking out gene function, this technique can also identify partial loss-of-function alleles or other alterations of gene function that can be extremely useful for investigating the function of essential genes or genes encoding proteins with multiple domains. A well-known example illustrating the value of an allelic series of mutations is the elucidation of the functions of the *let-60 *gene of *C. elegans *which encodes a member of the GTP-binding RAS proto-oncogene family involved in signalling (reviewed in [49]. Different mutations in this gene can have recessive, semi-dominant, or dominant phenotypes that define functions for the protein in developmental processes as diverse as vulval induction, migration of the sex myoblasts, function of chemosensory neurons, progression through pachytene in meiosis I, and differentiation of the excretory cell. Thus, a comprehensive understanding of the biology of a given gene is often revealed using non-null mutations. In this study we have identified 42 new missense mutations and two nonsense mutations that are available for genetic studies, and preliminary analysis indicates that at least some of these have deleterious effects on phenotype.

## Conclusions

We have used TILLING in *C. elegans *to determine the spectrum of mutations induced by EMS in this species and found that 96% of the mutations we identified were G/C-to-A/T transitions. In this pilot project we identified 71 point mutations in 10 genes, of which 44 or more may have an effect on gene function. For seven of the genes we targeted no mutant strains were previously available from the *Caenorhabditis *Stock Centre. One of the remaining target genes had a deletion mutation, but the strain carrying this mutation was shown to also carry a wildtype copy of the gene. Hence, for eight of the 10 target genes screened, TILLING has provided the first genetically heritable mutations which can be used to study their functions *in vivo*.

A frozen library of more than 1500 EMS-mutagenised worms was constructed, and enough DNA has been extracted and purified to screen for mutations in more than 5000 genes. The initial construction of the mutant library is labour-intensive but, if well-constructed, it should only be necessary to perform this step once. Approximately one gene, per Li-cor sequencer, per week can be screened after library construction is complete. The cost and rate of TILLING is dependent partially on the quality of the DNA being screened (how reliably the reactions work) and the mutation rate (how many alleles are identified per pooled population). Current estimates of cost-per-gene vary from $1500 to $2500 USD, including equipment, labour and consumables.

TILLING appears to provide a reasonably high proportion of missense mutations in *C. elegans *probably, in part, because of the small size of *C. elegans *introns compared to some other species. With EMS, the expected frequency of nonsense and splice junction mutations from TILLING screens is approximately five percent in most species. The two putative null alleles of this type that we have identified (out of 71 mutations) represent a frequency not significantly different from the expected five percent. At the CAN-TILL facility TILLING is successfully being used in many species for the detection of both induced and natural variation (reviewed in [50]) and there appears to be no species bias in terms of performance. It would seem, however, to be especially useful for genetically tractable organisms such as *C. elegans *where genomics tools are well developed, but where reverse genetics techniques that can provide heritable mutations suitable for genetic analysis lag far behind.

## Methods

### Mutagenesis and library construction

N2 wild type hermaphrodites were exposed to 0.025 M EMS for 4 h (as in Brenner, 1974 [51], but at a lower EMS concentration). After exposure to mutagen, the worms were washed and allowed to recover for 1–2 h before young gravid hermaphrodites were picked to fresh plates (10 per plate). After 24 h, the mutagenised P_0 _animals were transferred to fresh plates for a second 24 h brood. F_1 _progeny were picked 1 per plate and allowed to self-fertilize. The F_1 _progeny of mutagenised worms were set up individually rather than in pooled populations so as to ultimately simplify the isolation of F_3 _animals carrying any mutations. This was considerably more work than would have been required with pooled F_1 _samples but, because worm libraries were frozen, the extra work was a one time occurrence and greatly simplified identification of thawed mutants. When the mutagenised worm populations had exhausted the bacteria on the plate, worms were washed off each plate with 2 ml of M9 buffer. One third of the population from each F_1 _line was used for DNA and the other two thirds was frozen for future use as follows: from each plate, 0.5 ml of worms were mixed with 0.5 ml of 2X freeze solution (100 mM NaCl, 50 mM KPO_4 _pH 6.0, 0.3 mM MgSO_4, _30% glycerol) and frozen at – 80°C to generate a frozen library stock.

The remaining 1 ml of worms in M9 buffer for each line was centrifuged at 14,000 g to pellet worms. Excess buffer was aspirated leaving approximately 50 μl of buffer and worms in each tube. 50 μl of worm lysis buffer (50 mM KCl, 10 mM Tris (pH 8.3), 2.5 mM MgCl2, 0.45% Nonidet P-40, 0.45% Tween 20, 0.01% (w/v) gelatin, and 10 mg/ml proteinase K) was added to each tube which was then frozen at -80°C for at least 1 h. DNA was extracted by proteinase K lysis at 57°C for 4 h with occasional vortexing, and then 100 μl of phenol:chloroform:isoamyl alcohol (24:24:1) solution was added to the crude DNA extracts and tubes were vortexed for 5 minutes. Phases were separated by centrifugation at 14,000 g for 5 minutes and then aqueous layer was removed to a fresh tube containing 100 μl of chloroform, vortexed for 5 minutes and the phases separated by centrifugation at 14,000 g. The aqueous layer was again removed to a fresh tube containing 400 μl of isopropanol, and then the DNA was precipitated by centrifugation at 14,000 g for 10 minutes. DNA pellets were washed once with 70 % ethanol, resuspended in 50 to 100 μl ddH_2_O and quantified using a ND-1000 Spectrophotometer (NanoDrop Technologies, Wilmington, DE, USA). Samples were diluted to 1 ng/ul in 10 mM Tris, 1 mM EDTA pH 7.4, and 1 ml aliquots were distributed in plates of 64 samples (8 rows by 8 columns) before pooling. The DNA samples were then pooled 8-fold, in both column and row directions, and then distributed into 96-well plates of 8-fold column pools and 96-well plates of 8-fold row pools for each library of 768 individuals.

### Primer design and PCR Amplification

Primers were designed, using the web-based programme CODDLE [39] and selecting "EMS (not TILLING)" as the Mutation Method since a *C. elegans *option was not available and we did not know how similar the spectrum of mutations caused by EMS was in *C. elegans *compared to other organisms. Primers for C05C10.5 were designed to amplify a fragment of approximately 1200 bp since the genomic size of this gene is only 788 bp. The other primer sets were designed to amplify fragments as close to 1500 bp as possible, given the structure of the DNA in the region. Primers were purchased from MWG Biotech, Inc. (High Point, NC, USA), suspended to a concentration of 100 uM in 10 mM Tris, 1 mM EDTA pH 7.4 and used at a final concentration of 0.2 mM in a mixture of 3:2 (labeled:unlabeled) for the forward (IRD700-labeled) primers and 4:1 (labeled:unlabeled) for the reverse (IRD800-labeled) primers as per Colbert *et al*., 2001 [52]. PCR was also performed according to Colbert *et al*.,2001 [52]: 10 ul PCR reactions with 2.5 ng – 5 ng of genomic DNA were used for amplification in 96-well or 384-well PCR plates using ExTaq polymerase (Takara Bio Inc, Japan), but with 0.6 times the recommended concentration of ExTaq buffer and 2 mM MgCl_2_. PCR cycles were as follows: 95°C for 2 min; eight cycles of [94°C for 20 sec, 73°C for 30 sec (decrementing 1°C per cycle), 72°C for 1 min]; 45 cycles of: [94°C for 20 sec, 65°C for 30 sec, and 72°C for 1 min]; 72°C for 5 min; 99°C for 10 min (denaturation and inactivation of taq enzyme); and 70 cycles of 20 sec at 70°C (decrementing 0.3°C per cycle for random reannealing to allow hybridisation of mutant and wildtype molecules), hold at 4°C.

### Preparation of celery juice extract

Crude celery juice extract (CJE) was prepared as described by Till *et al*. [53]. Briefly, 0.5 kg of celery was processed in a kitchen-quality juicer until liquefied. Tris HCl (pH 7.7) was added to 0.1 M along with Phenylmethylsulphonylfluoride (PMSF) to 100 mM. The solution was spun at 2600 G for 20 minutes and the supernatant removed, brought to 25% saturation in (NH_4_)_2_SO_4_, mixed for 30 minutes at 4°C, and spun at 15,000 G for 40 minutes at 4°C. The supernatant was removed again and adjusted to 80% saturation in (NH_4_)_2_SO_4_, mixed for 30 minutes at 4°C, and spun at 15,000 G for 1.5 hours at 4°C. The pellet from this cut was resuspended in 1/10 the starting volume of 0.1 M Tris HCl (pH 7.7), 100 mM PMSF. The suspension was dialysed against 8 L of the same buffer, four times, for one hour each time at 4°C using Spectrapore dialysis tubing (10,000 MW cut-off). Aliquots were stored at -70°C and were spun at approximately 2000 G for one minute before use to remove any tissue debris.

### CJE digestion, sequence analysis and identification of mutants

PCR products were digested with CJE by adding 20 μl of extract and buffer mix (100 mM MgSO4, 100 mM HEPES, 300 mM KCl, 0.02% Triton X-100, 0.002 mg/ml BSA, and 0.2% to 0.3% crude CJE) directly to the PCR reactions and incubating at 45°C for 15 minutes. Reactions were stopped by adding 2.5 μl of 0.5 M EDTA. The DNA was purified by passage through G50 Sephadex in 96-well Millipore Multiscreen^® ^filtration plates (Millipore Corporation, Billerica, MA) and concentrated for 30 minutes at 90°C before running on a 25 cm long LI-COR acrylamide gel with a 0.4 mm wide, 96-well sharkstooth comb. Analysis of the gel images was done using GelBuddy [54] to define lanes and estimate sizes of cleavage products. The correlation of row and pool columns indicated which individual F_1 _worm carried the mutation. Most mutations were sequenced in both directions using either the same forward or reverse primers as for PCR or an internal primer designed for sequencing. Sequence analysis was performed using Sequencher 4.2 (Gene Codes Corporation, Ann Arbor, MI, USA) and the potential effect of the mutations was predicted using PARSESNP [42].

### Pooling

DNA from 4 strains previously shown to carry a single mutation each was screened to test the efficiency of different pooling depths. Four bands were expected in each of the two channels of the Li-cor sequencing image when amplified DNA from these individuals was run on a gel. With 4-fold pooling all eight mutant bands were detected (four in the 700 channel image and four in the 800 channel image), as they were with the 8-fold pools, whereas with 12-fold pooling, six of the eight mutant bands were seen on one test gel (three in the 700 channel image and three in the 800 channel image), and only three bands on the second gel (two in the 700 channel image and one in the 800 channel image). Based on these data and data from other studies, we conclude that 8-fold pooling was the best option for *C. elegans*.

### Identification of mutant individuals

Frozen worms were thawed and 20 individuals were plated 1 per 60 mm NGM plate and allowed to self-fertilize. Two approaches were taken to identify lines containing the target mutation. In the first approach, the 20 individuals from the strain carrying the mutation of interest were plated individually, allowed to grow until plates were starved, and DNA was prepared as described for the DNA library construction. In the second approach, individual animals from the strain carrying the mutation of interest were allowed to lay eggs for 2–3 days, and then the parent was picked into 5 μl of lysis buffer and lysed at 57 °C for 1 h followed by 95 °C for 15 m to inactivate the proteinase K. In both approaches the extracted DNA was PCR amplified and the mutation detected either by TILLING, or by direct sequencing of the amplified DNA, or by digestion of the PCR product with restriction enzymes resulted in a banding pattern different from wild type. Restriction enzyme site changes were found by PARSESNP to occur in 60 of 71 mutations.

If homozygous lines were not identified, 20 more individuals were set up from one line that had been shown to be heterozygous and the progeny from each of these lines was screened for evidence of deleterious mutations that might result in inviability. If sterile adults, dead embryos or larval lethals were observed, DNA was prepared from these and tested for the presence of the targeted mutation. Mutations that segregated with inviable phenotypes were balanced with genetic balancers to prevent the loss of the mutation.

### Statistical comparison of results from TILLING in different organisms

Frequencies of EMS-induced mutations identified during different TILLING experiments have been based on different samples sizes in different species. When comparing our data with others we used an on-line Proportions Test [55] to compare the frequencies we observed with those reported in other species and determine whether or not observed differences were significant (at the 90% confidence level) or were simply likely to be the result of sample size differences.

## List of abbreviations used

bp: base pair(s);

CAPS: cleaved amplified polymorphic sequences;

CJE: celery juice extract;

dCAPS: derived cleaved amplified polymorphic sequences;

dsRNA: double stranded RNA;

EMS: ethylmethanesulfonate;

RNAi: RNA interference;

TILLING: Targeting Induced Local Lesions in Genomes;

TMP/UV: trimethylpsoralen and ultraviolet radiation

## Authors' contributions

EJG and MCZ conceived of the study. GWH participated in its design and coordination. AMR and NJO carried out the mutagenesis and culturing of nematode strains. EJG performed the TILLING and data analysis and wrote the manuscript. MCZ and NJO analysed mutant phenotypes. All authors read and approved the final manuscript.

## Supplementary Material

Additional File 1 Table in MS Word that shows a list of mutations identified in forward genetic screens of EMS-treated *C. elegans *(obtained from Wormbase [7]). Click here for file

## Figures and Tables

**Table 1:** List of TILLING targets, sizes of amplicons and number and type of mutations identified for each gene.

**Gene Name**	**Description**	**Gene Size (bp)**	**PCR Size (bp)**	^1^**Prev. alleles**	^2^**Prev. strains**	^3^**Mis- sense**	^3^**Null**	^3^**Silent**	^3^**Total**
*C05C10.5	Hypothetical protein	788	1175	0	0	2		1	3
*mel-32 *C05D11.11	Serine hydroxyl-methyl-transferase	1600	1500	16	1	4	1	2	7
*mus-81 *C43E11.2	Endonuclease MUS81	2530	1171	1	0	4		3	7
*xpf-1 *C47D12.8	Structure-specific endonuclease ERCC1-XPF	9112	1452	0	0	5		3	8
*F25H2.13	Helicase of the DEAD superfamily	4985	1499	1	0	5		4	9
*htp-3 *F57C9.5	HIM-3 paralogue	2598	1452	1	1	5		5	10
*M03C11.2	Helicase of the DEAD superfamily	5943	1490	1	0	4		1	5
*cki-2 *T05A6.2	Hypothetical protein	1555	1569	0	0	7	1	2	10
*mdf-2 *Y69A2AR.30	Spindle assembly checkpoint protein	4461	1466	1	0	1		5	6
*htp-2 *Y73B6BL.2	HIM-3 paralogue protein 2	1199	1451	0	0	5		1	6
**Totals**			**14225**			**42**	**2**	**27**	**71**

* Gene name not assigned

^1 ^Number of mutant alleles listed in Wormbase [7] as existing prior to this study.

^2 ^Number of mutant strains available from the Caenorhabditis Genetic Stock Center.

^3 ^Number of mutations of this type identified in this TILLING study.

Missense mutations alter the amino acid sequence of the encoded protein. Null mutations refer to mutations that convert an amino acid codon into a premature stop codon, or that alter a conserved splice junction and result in premature truncation of the protein product of the gene. Silent mutations are changes that do not affect the protein product of the gene. These include mutations in introns or intergenic sequences, and mutations that alter the third bp of a codon in such a way that it does not change the amino acid encoded by that codon.

**Table 2:** Mutations identified by TILLING.

**Gene**	**Strain**	**Allele**	**Change**	**Effect**
C05C10.5	CN556	vc21	C112T	P23S
C05C10.5	CN1688	vc40	G178A	G45R
C05C10.5	CN1746	vc41	G230A	Non-coding
*mel-32 *C05D11.11	CN843	vc11	G361A	V106I
*mel-32 *C05D11.11	CN1181	vc68	C1339T	Q416*
*mel-32 *C05D11.11	CN1621	vc69	G289A	G82R
*mel-32 *C05D11.11	CN1665	vc70	G373A	D110N
*mel-32 *C05D11.11	CN1738	vc71	G384A	K113=
*mel-32 *C05D11.11	CN1805	vc72	G972A	K293=
*mel-32 *C05D11.11	CN1856	vc73	G373A	D110N
*mus-81 *C43E11.2	CN1162	vc42	C1830T	L368F
*mus-81 *C43E11.2	CN1162	vc43	G1231A	Non-coding
*mus-81 *C43E11.2	CN1211	vc44	A955T	Non-coding
*mus-81 *C43E11.2	CN1456	vc45	G972A	Non-coding
*mus-81 *C43E11.2	CN1604	vc46	G1897A	G390E
*mus-81 *C43E11.2	CN1766	vc47	C1687T	T320I
*mus-81 *C43E11.2	CN568	vc48	G1313A	D214N
*xpf-1 *C47D12.8	CN665	vc18	C602T	L183F
*xpf-1 *C47D12.8	CN720	vc19	G930A	R292H
*xpf-1 *C47D12.8	CN1286	vc62	G1036A	R278Q
*xpf-1 *C47D12.8	CN1475	vc63	G446A	E100K
*xpf-1 *C47D12.8	CN1574	vc64	C101T	Non-coding
*xpf-1 *C47D12.8	CN1574	vc65	G818T	S205=
*xpf-1 *C47D12.8	CN1751	vc66	C902T	V233=
*xpf-1 *C47D12.8	CN1798	vc67	G942A	D247N
F25H2.13	CN838	vc10	A1200T	I277F
F25H2.13	CN1245	vc52	G1206A	E279K
F25H2.13	CN1326	vc53	G421A	Non-coding
F25H2.13	CN1742	vc54	G285A	E95=
F25H2.13	CN1812	vc55	G399A	Non-coding
F25H2.13	CN1838	vc56	G1167A	A266T
F25H2.13	CN579	vc7	C649T	Non-coding
F25H2.13	CN579	vc8	C1165T	S265F
F25H2.13	CN48	vc9	G1242A	E291K
*htp-3 *F57C9.5	CN646	vc1	G2224A	E616K
*htp-3 *F57C9.5	CN823	vc13	G1785A	E469=
*htp-3 *F57C9.5	CN727	vc2	G2029A	E551K
*htp-3 *F57C9.5	CN1362	vc23	C2048T	P557L
*htp-3 *F57C9.5	CN1369	vc24	G1905A	S509=
*htp-3 *F57C9.5	CN1425	vc25	G2181A	Q601=
*htp-3 *F57C9.5	CN1630	vc26	G2230A	V618I
*htp-3 *F57C9.5	CN1723	vc27	C1245T	P306L
*htp-3 *F57C9.5	CN1735	vc28	C2331T	Y651=
*htp-3 *F57C9.5	CN825	vc3	G1333A	R335=
M03C11.2	CN1246	vc57	G4804A	E680K
M03C11.2	CN1479	vc58	G5097A	G725D
M03C11.2	CN1543	vc59	C4755T	I663=
M03C11.2	CN1643	vc60	C4739T	P658L
M03C11.2	CN1712	vc61	C5740T	H782Y
*Cki-2 *T05A6.2	CN843	vc20	C413T	T123I
*Cki-2 *T05A6.2	CN1157	vc31	G869A	Non-coding
*Cki-2 *T05A6.2	CN1231	vc32	C338T	T98I
*Cki-2 *T05A6.2	CN1254	vc33	G214A	G57R
*Cki-2 *T05A6.2	CN1309	vc34	G524A	E146K
*Cki-2 *T05A6.2	CN1364	vc35	G876A	Non-coding
*Cki-2 *T05A6.2	CN1575	vc36	G370A	V109M
*Cki-2 *T05A6.2	CN1643	vc37	C170T	S42F
*Cki-2 *T05A6.2	CN1672	vc38	G148A	E35K
*Cki-2 *T05A6.2	CN1787	vc39	G76A	Splice Junction
*mdf-2 *Y69A2AR.30	CN711	vc15	G243A	D65N
*mdf-2 *Y69A2AR.30	CN902	vc17	C1083T	Non-coding
*mdf-2 *Y69A2AR.30	CN1613	vc49	C838T	Non-coding
*mdf-2 *Y69A2AR.30	CN1703	vc50	C838T	Non-coding
*mdf-2 *Y69A2AR.30	CN1865	vc51	C520T	Non-coding
*mdf-2 *Y69A2AR.30	CN1114	vc74	G76A	Non-coding
*htp-2 *Y73B6BL.2	CN750	vc14	C507T	A139V
*htp-2 *Y73B6BL.2	CN50	vc22	C756T	T222I
*htp-2 *Y73B6BL.2	CN1271	vc29	C712T	S207=
*htp-2 *Y73B6BL.2	CN1540	vc30	G345A	R85Q
*htp-2 *Y73B6BL.2	CN574	vc5	G49A	D17N
*htp-2 *Y73B6BL.2	CN901	vc6	G878A	G263R

One letter nucleotide and amino acid codes follow IUPAC-IUB nomenclature. The first letter in the Nucleotide Change column indicates the wildtype nucleotide at this site, followed by the position of the mutation from the start codon in the genomic DNA and then the mutant nucleotide. The first letter in the Effect column indicates the wildtype amino acid at this site, followed by the position of the mutation within the predicted protein sequence and then mutated amino acid. An equal sign after the amino acid position means no change in the amino acid encoded by that codon, and an asterisk indicates a stop codon. Mutations in introns and intergenic regions are designated "Non-coding".

**Table 3:** Primers used to amplify target genes in pilot *C. elegans *TILLING project

**Gene**	**Oligo Name**	**Oligo Sequence**
F57C9.5	ce0001Lb	GTGCTGAGAATCCTGAACTTGACG
	ce0001R	TCTACTTGGCATGTTCGGCGACTG
Y73B6BL.2	ce0002L	GGGTTCGCGAATTTCACTTGCATT
	ce0002R	CGGCTCCTCTGCGAGTAGTTGGTC
T05A6.2	ce0003L	GCGGCGCACTCACATTTTTCTCTT
	ce0003R	CTGTGCGGACTTTGGCACATTTGA
C05C10.5	ce0004L	GAACTATTTGTGCGCGCGCGTTT
	ce0004R	TCAATGAGTGGGGTGGATTCAAGAAGA
C43E11.2	ce0006-3L	CTCCGAAATGAGAACTGTCCGACCAAT
	ce0006-3R	AAAGCTGAAGAAGTCGAATCGGTGCAT
Y69A2AR.30	ce0007L	CGCGATTTCCCTCAAAGGATCTGC
	ce0007R	AGAGCACCATCACACCACCTGACG
F25H2.13	ce0008L	TCAAAAAGAGACGAAGCCGCTGGA
	ce0008R	GCAGCAGCAACATCTTGAGCGTGT
M03C11.2	ce0009-2L	CAGCTCAGCTTCTCGTGGAGACCCTAT
	ce0009-2R	AGGAATCTTTAGAGCAACCGGGCAAAA
C47D12.8	ce0010L	CCGGAATCGCATTGATTCCAAAAG
	ce0010R	TGCAGCGAAATCACTTACAATCGTTTCC
C05D11.11	ce0011L	CGCCACAAGTACACCAACAACGAGAA
	ce0011R	GCGAGATCAGCGACGTCTTTCTTGA
