# Supplementary material for: TILLING is an effective reverse genetics technique for Caenorhabditis elegans
Source: BMC Genomics. 2006 Oct 18;7:262. doi: 10.1186/1471-2164-7-262 (PMC1626091; doi:10.1186/1471-2164-7-262)
Supplement: Additional File 1 — Table in MS Word that shows a list of mutations identified in forward genetic screens of EMS-treated C. elegans (obtained from Wormbase [7]). [file 1471-2164-7-262-S1.doc]

**Additional File 1**

List of mutations identified in forward genetic screens of EMS-treated *C. elegans* (obtained from [http://www.wormbase.org](http://www.wormbase.org/)).

| **Gene** | **EMS allele** | **Sequence** | **Effect** | **Notes** |
| --- | --- | --- | --- | --- |
|  |  |  |  |  |
| anc-1 | e1873 | c/t | nonsense |  |
| atp-2 | ua2 | 710 bp deletion | |  |
| ceh-2 | ch4 | 2314 bp deletion | |  |
| dpy-11 | e207 | c/t | nonsense |  |
| dpy-11 | e224 | g/a | missense |  |
| dpy-11 | e33 | g/a | nonsense |  |
| dpy-17 | e164 | c/t | nonsense |  |
| dpy-22 | bx103 | g/a | splice site |  |
| dpy-22 | sy622 | c/t | nonsense |  |
| dpy-22 | sy665 | c/t | nonsense |  |
| egl-13 | n483 | c/t | missense |  |
| egl-15 | n1457 | g/a | nonsense |  |
| egl-15 | n1459 | g/a | splice site |  |
| egl-15 | n1460 | g/a | splice site |  |
| egl-15 | n1475 | g/a | nonsense |  |
| egl-15 | n1476 | g/a | missense |  |
| egl-15 | n1477 | g/a | nonsense |  |
| egl-15 | n1775 | g/a | missense |  |
| egl-15 | n1780 | g/a | missense |  |
| egl-15 | n1783 | c/t | missense |  |
| egl-15 | n1784 | g/a | splice site |  |
| egl-15 | n2182 | c/t | missense |  |
| egl-15 | n2184 | g/a | missense |  |
| egl-15 | n2189 | c/t | missense |  |
| egl-15 | n2202 | g/a | missense |  |
| egl-15 | n2203 | g/a | missense |  |
| egl-15 | n2205 | g/a | missense |  |
| egl-15 | n2206 | g/a | nonsense |  |
| egl-15 | n2210 | g/a | missense |  |
| egl-18 | ga97 | c/t | nonsense |  |
| egl-18 | n162 | c/t | nonsense |  |
| egl-18 | n474 | single bp deletion | |  |
| egl-18 | n475 | a/t | nonsense |  |
| egl-20 | mu27 | g/a | missense |  |
| egl-20 | mu39 | g/a | missense |  |
| egl-20 | n585 | g/a | missense |  |
| egl-21 | n476 | 88 bp deletion | |  |
| egl-21 | n576 | g/a | splice site |  |
| egl-26 | ku211 | g/a | ? |  |
| egl-3 | n150 | g/a | missense |  |
| egl-3 | n588 | g/a | missense |  |
| egl-3 | n589 | g/a | missense |  |
| egl-4 | ks60 | 958 bp deletion | |  |
| egl-4 | ks61 | c/t | nonsense |  |
| egl-4 | ks62 | g/a | nonsense |  |
| egl-4 | n478 | g/a | missense |  |
| egl-4 | n479 | c/t | nonsense |  |
| egl-4 | n612 | g/a | missense |  |
| egl-47 | n1081 | c/t | missense |  |
| egl-47 | n1082 | c/t | missense |  |
| egl-8 | n488 | 1819 bp deletion | |  |
| let-60 | ay75 | g/a | missense |  |
| lin-1 | e1026 | c/t | nonsense |  |
| lin-1 | n176 | c/t | nonsense |  |
| lin-1 | n303 | g/a | missense |  |
| lin-14 | n531 | g/a | splice site |  |
| lin-14 | n534 | g/a | nonsense |  |
| lin-23 | ot1 | c/t | missense |  |
| lin-26 | mc1 | g/a | missense |  |
| lin-26 | mc2 | c/t | missense |  |
| lin-26 | mc4 | g/a | nonsense |  |
| lin-26 | n156 | c/t | missense |  |
| lin-31 | ga10 | 957 bp deletion | |  |
| lin-31 | ga57 | a/t | missense |  |
| lin-31 | ga70 | c/t | nonsense |  |
| lin-31 | n1048 | c/t | nonsense |  |
| lin-31 | n1053 | g/a | nonsense |  |
| lin-31 | n2520 | c/t | missense |  |
| lin-31 | n301 | g/a | missense |  |
| lin-31 | n376 | 536 bp deletion | |  |
| lin-31 | n435 | c/t | nonsense |  |
| lin-31 | n762 | g/a | nonsense |  |
| lin-31 | oz201 | g/a | missense |  |
| lin-9 | n112 | g/a | missense |  |
| lin-9 | n942 | g/a | nonsense |  |
| spn-4 | or191 | g/a | missense |  |
| spn-4 | or25 | g/a | missense |  |
| sup-9 | e2655 | g/a | missense |  |
| sup-9 | n1008 | c/t | nonsense |  |
| sup-9 | n1009 | g/a | missense |  |
| sup-9 | n1012 | c/t | nonsense |  |
| sup-9 | n1016 | t/a | missense |  |
| sup-9 | n1017 | g/a | nonsense |  |
| sup-9 | n1020 | g/a | missense |  |
| sup-9 | n1023 | c/t | nonsense |  |
| sup-9 | n1025 | c/t | missense |  |
| sup-9 | n1026 | g/a | splice site |  |
| sup-9 | n1028 | g/a | splice site |  |
| sup-9 | n1037 | c/t | nonsense |  |
| sup-9 | n1549 | g/a | nonsense |  |
| sup-9 | n1550 | g/a | missense |  |
| sup-9 | n1553 | g/a | splice site |  |
| sup-9 | n1557 | t/c | missense |  |
| sup-9 | n186 | c/t | nonsense |  |
| sup-9 | n190 | c/t | missense |  |
| sup-9 | n191 | g/a | missense |  |
| sup-9 | n213 | g/a | missense |  |
| sup-9 | n2174 | g/a | splice site |  |
| sup-9 | n2175 | g/a | splice site |  |
| sup-9 | n2176 | g/a | missense |  |
| sup-9 | n2276 | g/a | nonsense |  |
| sup-9 | n2278 | c/t | missense |  |
| sup-9 | n2279 | g/a | splice site |  |
| sup-9 | n2281 | c/t | missense |  |
| sup-9 | n2282 | g/a | missense |  |
| sup-9 | n2283 | g/a | missense |  |
| sup-9 | n2284 | 561 deletion | |  |
| sup-9 | n2285 | g/a | splice site |  |
| sup-9 | n2286 | g/a | missense |  |
| sup-9 | n2287 | 155 deletion | |  |
| sup-9 | n2288 | g/a | missense |  |
| sup-9 | n2291 | a/t | missense |  |
| sup-9 | n2292 | g/a | nonsense |  |
| sup-9 | n2294 | g/a | missense |  |
| sup-9 | n2296 | g/a | missense |  |
| sup-9 | n2297 | g/a | nonsense |  |
| sup-9 | n2343 | c/t | missense |  |
| sup-9 | n2344 | g/a | missense |  |
| sup-9 | n2345 | c/t | missense |  |
| sup-9 | n2346 | g/a | missense |  |
| sup-9 | n2347 | c/t | missense |  |
| sup-9 | n2348 | g/a | missense |  |
| sup-9 | n2349 | g/a | missense |  |
| sup-9 | n2350 | g/a | missense |  |
| sup-9 | n2351 | g/a | missense |  |
| sup-9 | n2352 | a/t | missense |  |
| sup-9 | n2353 | c/t | missense |  |
| sup-9 | n2354 | g/a | missense |  |
| sup-9 | n2355 | g/a | missense |  |
| sup-9 | n2356 | c/t | missense |  |
| sup-9 | n2357 | g/a | nonsense |  |
| sup-9 | n2358 | c/t | missense |  |
| sup-9 | n2359 | g/a | missense |  |
| sup-9 | n264 | c/t | missense |  |
| sup-9 | n266 | g/a | nonsense |  |
| sup-9 | n271 | g/a | splice site |  |
| sup-9 | n3310 | g/a | missense |  |
| sup-9 | n508 | c/t | missense |  |
| sup-9 | n619 | g/a | missense |  |
| sup-9 | n659 | g/c | splice site |  |
| sup-9 | n668 | c/t | nonsense |  |
| tbb-2 | or362 | g/a | missense |  |
| tbb-2 | sb26 | g/a | missense |  |
| tbb-2 | t1623 | g/a | missense |  |
| unc-10 | js244 | c/t | nonsense |  |
| unc-10 | ox120 | c/t | nonsense |  |
| unc-103 | e155 | c/t | nonsense |  |
| unc-103 | su142 | c/t | nonsense |  |
| unc-103 | su195 | g/t | nonsense |  |
| unc-119 | ed3 | c/t | nonsense |  |
| unc-119 | ed4 | c/t | nonsense |  |
| unc-119 | ed9 | g/a | splice site |  |
| unc-122 | n2916 | c/t | nonsense |  |
| unc-122 | ox79 | c/t | nonsense |  |
| unc-16 | e109 | 448 bp deletion | |  |
| unc-16 | ju146 | t/c | missense |  |
| unc-16 | ju79 | g/a | nonsense |  |
| unc-16 | n730 | g/a | nonsense |  |
| unc-2 | e55 | c/t | nonsense |  |
| unc-2 | ra605 | c/t | nonsense |  |
| unc-26 | e205 | g/a | nonsense |  |
| unc-32 | f121 | g/a | missense |  |
| unc-32 | f131 | g/a | splice site |  |
| unc-32 | n189 | c/t | missense |  |
| unc-38 | sy576 | c/t | missense |  |
| unc-5 | e152 | c/t | nonsense |  |
| unc-5 | e53 | g/a | nonsense |  |
| unc-5 | e553 | c/t | nonsense |  |
| unc-5 | e791 | c/t | nonsense |  |
| unc-52 | e1012 | c/t | nonsense |  |
| unc-52 | e1421 | g/a | splice site |  |
| unc-52 | e444 | c/t | nonsense |  |
| unc-52 | e669 | c/t | nonsense |  |
| unc-52 | e998 | g/a | nonsense | plus c/a 366 bp upstream |
| unc-52 | ra1 | g/a | splice site |  |
| unc-52 | ra12 | g/a | splice site |  |
| unc-52 | ra15 | g/a | splice site |  |
| unc-52 | ra16 | g/a | splice site |  |
| unc-52 | ra19 | g/a | splice site |  |
| unc-52 | ra22 | g/a | splice site |  |
| unc-52 | ra3 | g/a | splice site |  |
| unc-52 | ra37 | g/a | splice site |  |
| unc-52 | ra38 | g/a | missense | plus 311 bp deletion 326 bp upstream |
| unc-52 | ra4 | g/a | splice site |  |
| unc-52 | ra6 | g/a | splice site |  |
| unc-52 | ra7 | g/a | splice site |  |
| unc-52 | st549 | g/a | nonsense |  |
| unc-52 | su250 | c/t | intron |  |
| unc-53 | e2432 | 375 bp deletion | |  |
| unc-53 | n152 | 320 bp deletion | |  |
| unc-57 | e406 | single bp deletion | nonsense |  |
| unc-59 | e261 | g/a | missense |  |
| unc-60 | su158 | 698 bp deletion | |  |
| unc-61 | e228 | c/t | nonsense |  |
| unc-63 | x13 | g/a | nonsense |  |
| unc-63 | x26 | g/a | missense |  |
| unc-7 | hs10 | g/a | missense |  |
| unc-7 | hs9 | c/t | missense |  |
| unc-71 | e541 | g/a | missense |  |
| unc-71 | ju156 | 170 bp deletion | |  |
| unc-71 | ju157 | g/a | missense |  |
| unc-71 | ju159 | g/a | missense |  |
| unc-71 | ju160 | g/a | missense |  |
| unc-71 | ju161 | g/a | missense |  |
| unc-78 | e1217 | g/a | missense |  |
| unc-78 | e1221 | c/t | missense |  |
| unc-95 | su33 | c/t | nonsense |  |
| unc-97 | su110 | g/a | splice site |  |
| unc-98 | su130 | g/a | missense |  |
| zyg-1 | b1 | c/t | missense |  |
| zyg-11 | mn40 | c/t | nonsense |  |
